# Meaty Aroma Compounds Enhance MSG Umami Perception Through Allosteric Modulation of T1R1/T1R3 Receptor: Evidence from Nasal Clip Sensory Evaluation and Molecular Dynamics Simulation

**DOI:** 10.3390/foods14173041

**Published:** 2025-08-29

**Authors:** Yaqi Zhao, Jianan Zhang, Mouming Zhao, Xuan Zhao, Guowan Su

**Affiliations:** 1 School of Food Science and Engineering, South China University of Technology, No.381 Wushan Road, Tianhe District, Guangzhou 510640, China; zyq_viki@126.com (Y.Z.); femmzhao@scut.edu.cn (M.Z.); 2 National Engineering Research Center for Rice and By-Product Deep Processing, School of Food Science and Engineering, Central South University of Forestry and Technology, No.498 Shaoshan South Road, Changsha 410004, China; 3 College of Tropical Agriculture and Forestry, Guangdong AIB Polytechnic College, Guangzhou 510507, China; xuanzhao@gdaib.edu.cn

**Keywords:** umami enhancement, allosteric modulation, T1R1/T1R3 receptor, nasal clip evaluation, molecular dynamics simulation

## Abstract

Background: Understanding how aroma compounds enhance monosodium glutamate (MSG) umami perception remains a critical challenge in flavor science. Methods: The umami-enhancing effects of meaty flavorings were investigated using nasal clip sensory evaluation (orthonasal blockage). Active aroma compounds were subsequently identified using gas chromatography-mass spectrometry (GC-MS). The three-dimensional structure of the umami receptor T1R1/T1R3 was constructed by homology modeling. The interaction mechanism was deciphered using molecular dynamics (MD) simulations. Results: Seafood essence S demonstrated the most potent umami enhancement. Five key compounds significantly intensified the MSG umami intensity: methional, dimethyl sulfide (DMS), D-limonene (DLE), 2,3-dimethylpyrazine, and dimethyl trisulfide. Notably, this enhancement persisted even under nasal clip conditions, revealing a novel mechanism independent of cross-modal interactions. Sulfur-containing compounds consistently demonstrated umami-enhancing effects across the evaluation conditions. MD simulations showed that aroma compounds induced allosteric remodeling of T1R1/T1R3, strengthening MSG-receptor hydrogen bonding (1.8–2.6-fold increase), reducing receptor flexibility, and stabilizing the ternary complex. Binding affinity was highest for DMS, followed by DLE and methional. Conclusion: This study provides the first receptor-level evidence that aroma compounds directly modulate MSG-taste receptor interactions through allosteric regulation, offering a novel theoretical framework for odor–taste interactions with significant implications for umami enhancer design and flavor research.

## 1. Introduction

Flavor perception is not solely determined by the additive effects of taste compounds in food systems but emerges from complex interactions among flavor constituents [1,2]. Substantial evidence indicates that odors can enhance or suppress taste perception, particularly saltiness and sweetness [3,4,5,6,7]. Lawrence et al. [8,9] demonstrated through orthonasal and retronasal assessments that salty perception in low-concentration salt solutions could be potentiated by aroma compounds from specific foods (e.g., anchovy, bacon, smoked sardine, dry sausage, peanut). Djordjevic et al. [10] documented soy sauce odor-induced saltiness enhancement and strawberry odor-induced sweetness enhancement, confirming that imagined odors could modulate taste perception. Furthermore, umami perception, as one of the five basic tastes, is likewise influenced by food aromas. McCabe and Rolls [11] observed that combining a vegetable aroma with monosodium glutamate (MSG) yielded a more pleasant taste. Results of brain and magnetic resonance imaging revealed stronger activation in the medial orbitofrontal cortex and pregenual cingulate cortex during simultaneous odor-taste stimulation compared with summed separate stimuli. Niimi et al. [12] established that cheese aroma–taste interactions depended on taste type and aroma concentration. Specifically, the cheese aroma significantly intensified umami and bitterness but minimally affected sweetness, saltiness, or sourness. At low-to-medium concentrations, flavor intensity increased with higher MSG levels.

Flavor interactions encompass not only cross-modal sensory integration, but also the physicochemical interactions between substances including hydrogen bonding, hydrophobic bonding, ionic bonding, and covalent bonding. These interactions may influence both the mass transfer of flavor compounds in the oral cavity and their subsequent binding to receptors [13]. Odor-induced changes in taste perception (OICTP) involve multi-level mechanisms: at the neural level, they are manifested as mutual modulation of perceptual signals within the brain [10]; and at the molecular level, they originate from chemical reactions and physical interactions between the taste and odor compounds, leading to the binding of the resulting interaction products to taste receptors [14].

The mechanism underlying odor–taste interactions has constituted a core focus in flavor research. At the receptor level, prior studies have confirmed that aroma compounds can allosterically modulate the umami receptor T1R1/T1R3 [15], indicating that cross-modal interactions between olfaction and taste may originate within the peripheral taste system [16,17]. However, the precise pathway by which aroma compounds influence taste perception remains unclear: do these compounds directly bind to taste receptors, thereby altering receptor conformation, or do they indirectly modulate the binding of taste substances to their receptors? Elucidating these questions holds significant implications for refining flavor theory and clarifying the physiological mechanisms of odor–taste interactions.

Given the complexity of real food systems, where interaction mechanisms among multiple flavor constituents are challenging to elucidate precisely, this study employed a simplified model system with single compounds to investigate the mechanism underlying aroma-induced umami enhancement. Based on preliminary findings that meaty flavorings significantly potentiated umami perception, three commercial meaty flavorings (seafood essence S, beef essence B, and meat essence M) were selected as aroma sources to systematically examine their effects on MSG, which is the standard umami substance. This approach aimed to identify the key umami-enhancing aroma compounds and elucidate the molecular mechanism of aroma–umami interactions at the receptor level. To mitigate cross-modal interference from orthonasal olfaction and psychological confounders, nasal clip sensory evaluation was employed, enabling a more accurate reflection of the direct intermolecular interactions between flavor compounds.

This study established a technical pipeline: sensory screening → component identification → mechanistic investigation. Initially, meaty flavorings exhibiting significant umami-enhancing effects were screened via nasal clip sensory evaluation. Subsequently, volatile compound profiles within the potentiated flavorings were characterized using gas chromatography-mass spectrometry (GC-MS), with key umami-enhancing aroma compounds identified through sensory validation. Finally, the three-dimensional structure of the umami receptor T1R1/T1R3 was constructed by homology modeling, and molecular dynamics (MD) simulations were employed to decipher the regulatory effects of key aroma compounds on the binding process between MSG and the receptor, which encompassed binding site localization, binding energy alterations, and receptor conformational changes. Collectively, this work provides a theoretical foundation for understanding the molecular mechanism of aroma-induced umami enhancement.

## 2. Materials and Methods

### 2.1. Materials and Chemicals

Meat essence M (YR00359), beef essence B (YR01125), and seafood essence S (YR00315) were purchased from Givaudan Fragrances (Guangzhou) Co., Ltd. (Guangzhou, China). MSG was a food grade material and provided by Guangzhou Aosan MSG Food Co., Ltd. (Guangzhou, China). NaCl was provided by Guangdong Salt Group Co., Ltd. (Guangzhou, China). Food grade sugar was provided by Guangdong Zhongqing Sugar Group Co., Ltd. (Guangzhou, China). Citric acid monohydrate was provided by Weifang Ensign Industry Co., Ltd. (Changle, China). Quinine, dimethyl sulfide (DMS), and D-limonene (DLE) were purchased from Shanghai Aladdin Biochemical Technology Co., Ltd. (Shanghai, China). The 1-octen-3-ol, 3-methylthiopropionaldehyde (Methional), 2,3-dimethylpyrazine, 2,3,5-trimethyl pyrazine, isoamyl alcohol, dimethyl trisulfide, 2-methyl-3-heptanone, and C6-C33 n-alkane series were purchased from Sigma-Aldrich (St. Louis, MO, USA). All the other chemicals and solvents used were of analytical grade.

### 2.2. Taste Evaluation

#### 2.2.1. Panelist Training

The sensory evaluation was conducted according to the guidelines presented in the Declaration of Helsinki, and all panelists signed informed consent forms before the study. The panel consisted of 15 evaluators (4 males and 11 females, aged between 22 and 45 years old) from the School of Food Science and Engineering, South China University of Technology. The evaluators were selected from 46 volunteers, and through at least 1 year of sensory training. All participants were sensitive to the five basic tastes (umami, salty, bitter, sweet, and sour) and familiar with sensory evaluation. The screening and training process of the evaluators was mainly aimed at flavor discrimination ability and sensitivity. The investigation of taste discrimination ability was carried out through five basic taste matching experiments. The test solutions used were as follows: 0.3 g/L MSG solution for umami discrimination, 0.5 g/L salt solution for salty discrimination, 16 g/L sucrose solution for sweet discrimination, 0.03 g/L citric acid monohydrate solution for sour discrimination, and 0.003 g/L quinine solution for bitter discrimination. Volunteers who failed to identify the solution with the correct taste were eliminated. Next, evaluators were trained to familiarize themselves with the assessment scale through a ranking test. Different concentrations of MSG solution (0.7, 1.1, 1.8, 2.8 g/L), salt solution (1, 2, 3, 4 g/L), sucrose solution (1, 5, 10, 15 g/L), citric acid solution (0.05, 0.1, 0.2, 0.4 g/L), and quinine solution (0.001, 0.002, 0.003, 0.004 g/L) were prepared. With three random numbers, the samples were presented to the evaluators in the same order to ensure comparability. Each sample was tasted in the order from left to right, and the samples were arranged in the order of taste enhancement. Finally, the evaluator trained the sensitivity through the three-point test. The taste standard solutions were as follows: 0.1 g/L MSG solution, 0.5 g/L sodium chloride solution, 5 g/L sucrose solution, 0.01 g/L citric acid solution, and 0.001 g/L quinine solution. Three samples (taste standard solution or ultrapure water) formed a group, of which two were the same and the other one was different. After tasting, the one different from the other two samples was judged.

#### 2.2.2. Sensory Evaluation

Sensory evaluation of umami changes in MSG induced by odor were scored by the umami degree of the sample, with the 0.5 g/L MSG aqueous solution as 0 points and the 1.0 g/L MSG aqueous solution as 4 points. According to the degree of umami increase or decrease, that is, odor-induced changes in umami perception (OICUP), a vertical line was drawn at the corresponding position of the evaluation scale, −4~0 points (weaker umami), 0 points (equal umami), and 0~4 points (stronger umami). There were two types of sensory evaluation. One was no nasal clip sensory, which is conventional sensory evaluation. The other was nasal clip sensory evaluation, which refers to clamping the nose with a nasal clip during the evaluation. Nasal clip sensory evaluation excludes the influence of prenasal smell on flavor perception to a great extent.

In order to avoid the influence of sample appearance on the evaluation, a narrow glass bottle wrapped with tin foil paper was used to send three randomly numbered samples; furthermore, there was no more than five samples at a time. Different groups were assessed on different days. Sensory evaluation analysis was carried out in a sensory evaluation room at a temperature of 23 ± 2 °C, and 15 participants were professionally trained. The sensory evaluator kept about 2 mL of the sample in their mouth for about 2 s, moved the mouth, made the test solution fully in contact with the whole tongue, and then carefully identified the umami intensity before finally spitting out the sample solution. After each tasting, they were asked to rinse with 50–60 mL warm water, rest for 1 min, and then taste the next one.

#### 2.2.3. Nasal Clip Sensory Evaluation of Umami-Enhancing Meaty Flavorings on MSG

To evaluate the effect of the concentration of meaty flavoring on MSG umami perception, sample solutions were prepared with 0.125, 0.25, 0.5, 1, and 2 g/L of each essence based on the 0.5 g/L MSG solution and carried out by nasal clip sensory experiment. Then, in order to evaluate the effect of the concentration of MSG solution on meaty flavoring-induced umami changes in MSG, nasal clip sensory evaluation was executed by the essence with the concentration corresponding to the best umami enhance effect based on different concentrations of MSG solution (0.5, 1.0, and 3.0 g/L).

#### 2.2.4. Evaluation of Umami-Enhancing Aroma Compounds on MSG

The sample solution of aroma compounds was based on a 0.5 g/L MSG solution, and the corresponding concentration of aroma compounds in seafood essence S was added. To be specific, 1540 ppm for 1-octene-3-ol, 2580 ppm for DMS, 1630 ppm for methional, 1450 ppm for DLE, 1150 ppm for 2, 3-dimethylpyrazine, 7730 ppm for isoamyl alcohol, 1250 ppm for 2, 3, 5-trimethyl pyrazine, and 1130 ppm for dimethyl trisulfide. Each group of samples were carried out without a nasal clip and with a nasal clip sensory experiments, respectively.

### 2.3. Determination of Aroma Compounds in Meaty Flavorings

The experimental protocol was adapted, with appropriate modifications, from the procedure described by Cao et al. [18]. The GC-MS analyses were conducted on a Trace 1310 system (Thermo Fisher Scientific, Waltham, MA, USA) equipped with an ISQ LT mass-selective detector (Thermo Scientific, Waltham, MA, USA). Data acquisition and processing were performed using Xcalibur 4.1 software (Thermo Fisher Scientific, Waltham, MA, USA). A 0.05 g essence sample was placed into a 20 mL headspace vial with 20 μL of 2-methyl-3-heptanone (16.8 mg/L) used as the internal standard. The experimental conditions were set as follows: high-purity helium was employed as the carrier gas at a flow rate of 1.0 mL/min; an electron impact ionization (EI) source was used with an electron energy of 70 eV; the electron multiplier voltage was maintained at 350 V; the ion scanning range was 33~350 *m*/*z* with a scanning speed of 3.0 scans/s; and both the ion source temperature and transfer line temperature were set to 250 °C. For the solid-phase microextraction, the temperature program was as follows: the initial temperature was 40 °C held for 2 min, then increased to 120 °C at a rate of 5 °C/min and held for another 2 min, followed by a further ramp to 220 °C at 7 °C/min with a 5-min hold. The split ratio was set at 10:1. The retention indices (RIs) of the volatile compounds were calculated using a C6–C33 n-alkane series as the external standards under the same chromatographic conditions.

### 2.4. Homology Modeling Method of Umami Receptor T1R1/T1R3

The amino acid sequence of the umami receptor T1R1/T1R3 was obtained from the Uniprot website (https://www.uniprot.org/, accessed on 5 February 2021). The amino acid sequence of five crystal structures from a protein database (Protein data bank, PDB, https://www.rcsb.org/, accessed on 5 February 2021), 5K5S, 5X2M, 6N52, 5FBH, 2E4U, was compared with umami receptor T1R1/T1R3 (T1R1 UniProtKB: Q7RTX1, T1R3 UniProtKB: Q7RTX0) using NCBI’s Protein BLAST tool 2.11.0 [19].

Based on the three-dimensional structure file and sequence alignment file of the template protein, the similarity of the structure was inferred from the similarity of the sequence. The Modeller v9.19 program [20] was used to perform homology modeling on the umami receptor T1R1/T1R3 to obtain a reasonable three-dimensional structure model of the umami receptor, and the molecular mechanics optimization of the umami receptor model was performed. The structure of the umami receptor T1R1/T1R3 model was optimized by Amber14 force field [21]. The optimization process was carried out in two steps: the steepest descent method of 2000 steps was optimized first, and then the conjugate gradient method of 2000 steps was used to further optimize the structure. The final result was used as the model for subsequent analysis.

### 2.5. Molecular Dynamics (MD)

The MD simulation was performed using the Gromacs 2018.4 program [22] under constant temperature and pressure and periodic boundary conditions. Using the Amber99SB all-atom force field, during the MD simulation, all involved hydrogen bonds of TIP3P water model [23] were constrained by the LINCS algorithm [24] with an integral step of 2 fs. The electrostatic interaction was calculated using the PME (particle mesh Ewald) method [25]. The non-bond interaction cutoff value was set to 10 Å and updated every 10 steps.

The V-rescale temperature coupling method [26] was used to control the simulation temperature to 310 K, and the Parrinello–Rahman method [27] was used to control the pressure to 1 bar. First, the steepest descent method was used to minimize the energy of the two systems to eliminate the too close contact between the atoms. Then, a 100 ps NVT equilibrium simulation was performed at 310 K; finally, 50 ns MD simulations were performed on different systems, and the conformation was saved every 10 ps. The visualization of the simulation results was completed by the Gromacs embedded program and VMD.

### 2.6. Statistical Analysis

ANOVA and Duncan’s new multiple range test were performed to determine the significance of the differences between data at a 95% confidence interval using SPSS 14.0 software (SPSS Inc., Chicago, IL, USA). The OriginPro 2021 software (OriginLab Corp., Northampton, MA, USA) and Microsoft Office 2024 (Microsoft Corp., Redmond, Washington, DC, USA) were used for data visualization.

## 3. Results and Discussions

### 3.1. Effects of Meaty Flavorings on Umami Characteristics of MSG

**Figure 1:**
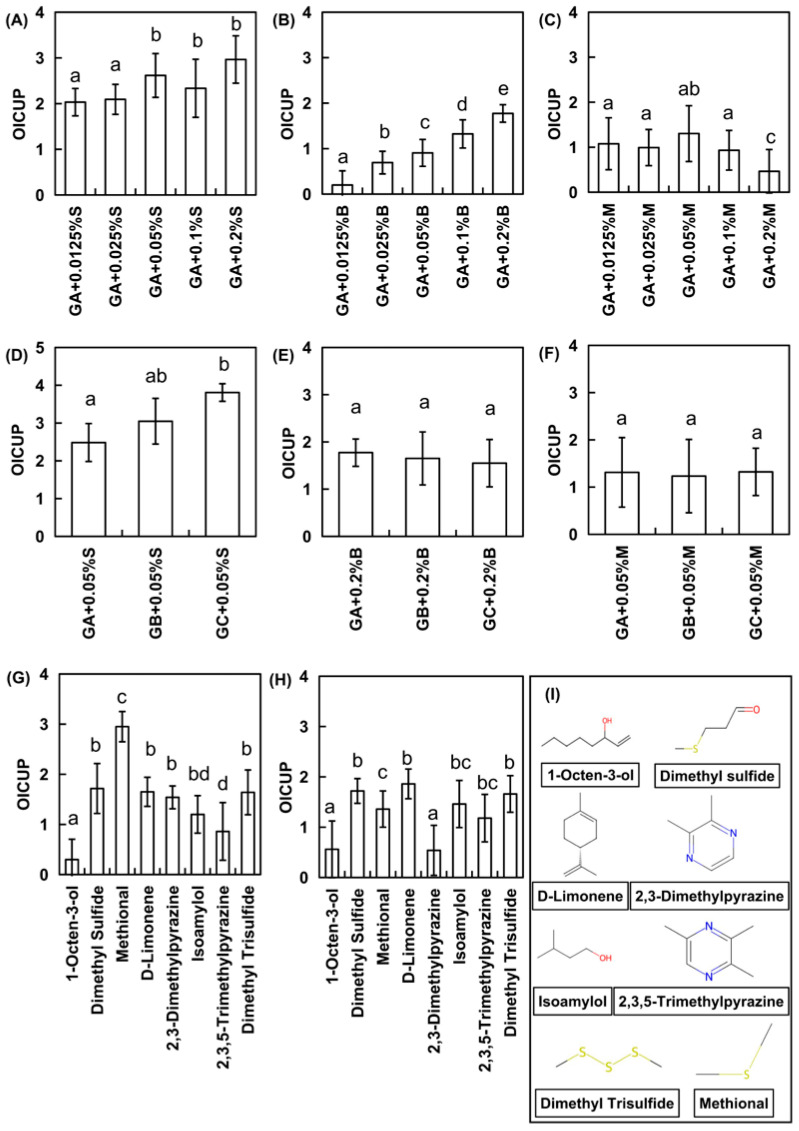
Odor induced changes in the umami perception (OICUP) of MSG by meaty flavorings and aroma compounds. (**A**–**C**) Effects of different concentrations of meat flavorings with nasal clip (**A**: seafood essence; **B**: beef essence; **C**: meat essence). (**D**–**F**) Effects of different MSG concentrations (GA: 0.5, GB:1.0, GC:3.0 g/L) enhanced by optimal flavoring concentrations with nasal clip. (**G**) Effect of individual aroma compounds on MSG umami perception. (**H**) Effect of individual aroma compounds on MSG umami perception with nasal clip. (**I**) Molecular structures of aroma compounds. Error bars represent standard deviation (*n* = 3). Different letters indicate significant differences (*p* < 0.05, Duncan’s test).

Three representative meaty flavorings—meat essence M, beef essence B, and seafood essence S—were selected to investigate their effects on MSG umami perception under nasal clip conditions (Figure 1). These flavorings were specifically procured as aroma-focused products. Prior to experimentation, analyses confirmed the absence of detectable non-volatile umami compounds (e.g., glutamic acid and disodium 5′-inosinate-5′-guanylate [I+G]) in all flavorings. The results revealed that seafood essence S exhibited the most pronounced umami enhancement, followed by beef essence B and meat essence M. This finding demonstrates marked variations in umami-enhancing efficacy among the flavoring types upon the elimination of orthonasal olfactory interference.

Further analysis indicated that all three flavorings exhibited marked concentration-dependent umami enhancement, though with divergent patterns (Figure 1). For seafood essence S (Figure 1A,D), the umami enhancement increased significantly over the 0.0125–0.1% concentration range. At higher concentrations (0.1–0.2%), the effects were more pronounced, but the incremental enhancement diminished. At the medium concentration (0.05%), significant enhancement was observed across all MSG levels, demonstrating greater efficacy at higher MSG concentrations. In beef essence B (Figure 1B,E), progressive umami enhancement occurred with increasing concentration. However, at the 0.2% concentration, enhancement effects converged across MSG levels with no significant differences observed. Regarding meat essence M (Figure 1C,F), the umami enhancement intensified at low-to-medium concentrations but declined at 0.2%, showing no significant variation across the MSG concentrations. This bell-shaped concentration–response pattern was consistent with the existing literature. Specifically, Niimi et al. [12] found that cheese aroma enhanced flavor at low-medium MSG concentrations but reduced it at high levels. Similarly, Nasri et al. [28,29] reported that the sardine aroma strengthened saltiness perception at low-to-medium salt concentrations, although its effects weakened under high-salt conditions.

Nasal clip sensory evaluation was conducted with physical nose occlusion to eliminate the orthonasal olfactory influence on flavor perception. This approach substantially mitigated cross-modal integration of taste and olfaction in the brain, enabling the direct interrogation of retronasal olfaction and intermolecular interactions on taste perception. Consequently, under conditions of suppressed orthonasal odor perception, seafood essence S demonstrated pronounced superiority in umami enhancement.

GC-MS analysis indicated significant compositional differences among the flavorings, as shown in Table 1. A total of 132 volatile compounds were identified in the three meaty flavorings. Sulfur-containing compounds, ketones, aldehydes, esters, and alkenes were identified as major chemical classes. For meat essence M, 47 volatile compounds were detected. Sulfur-containing compounds predominated including bis(2-methyl-3-furyl) disulfide, 2-pentylthiophene, 2-furanmethanethiol, 2-methyl-3-furanthiol, 2-furfuryl 2-methyl-3-furyl disulfide, ethyl octanoate, and 2-methyltetrahydrofuran-3-one. In beef essence B, 67 compounds were characterized. Alkenes exhibited the highest abundance including anethole, trans-caryophyllene, 4-allylanisole, trans-bergamotene, cis-α-bergamotene, copaene, zingiberene, octyl acetate, myristaldehyde, D-limonene, δ-elemene, (S)-β-bisabolene, 2-methyl-3-furanthiol, β-sesquiphellandrene, β-elemene, and α-caryophyllene. This terpenoid diversity likely resulted from the manufacturers’ blending practices with botanical flavor compounds to achieve complex sensory profiles. Regarding seafood essence S, 59 aroma compounds were identified. Sulfur compounds, esters, and aldehydes were prominent, with dimethyl disulfide, 1-octen-3-ol, dimethyl sulfide (DMS), 3-methylthiopropionaldehyde (methional), and D-limonene (DLE) occurring at high levels. Secondary constituents included 2,3-dimethylpyrazine, isoamyl alcohol, 2,3,5-trimethylpyrazine, (Z)-3-hexen-1-ol, dimethyl trisulfide, 2,3-diethylpyrazine, and 2-methylpyrazine. Ogasawara et al. [30] reported that the dried bonito aroma fraction containing sulfur compounds, pyrazines, alcohols, and phenols significantly enhanced the umami intensity across 0.68–1.5% salt concentrations. Thus, the substantial compositional variations among the flavorings likely underpin their differential efficacy in enhancing MSG umami perception. For instance, the research demonstrated that the specific odor-active compound, 3-mercapto-2-methylpentan-1-ol, could enhance the taste intensity of MSG [31].

Representative aroma compounds from seafood essence S, which were selected for their high abundance, broad applicability, and safety, were individually evaluated for umami-enhancing effects on MSG. The selected compounds were 1-octen-3-ol, DMS, methional, DLE, 2,3-dimethylpyrazine, 2,3,5-trimethylpyrazine, isoamyl alcohol, and dimethyl trisulfide. Their sensory evaluation results are presented in Figure 1G. A significant enhancement of MSG umami perception was demonstrated by methional, DMS, DLE, 2,3-dimethylpyrazine, and dimethyl trisulfide. Methional exhibited the most potent effect, while a moderate enhancement was observed for isoamyl alcohol, 2,3,5-trimethylpyrazine, and 1-octen-3-ol, with 1-octen-3-ol showing the weakest efficacy.

Under the nasal clip conditions (Figure 1H), the overall sensory intensity was reduced with diminished umami enhancement effects. DMS, DLE, and dimethyl trisulfide maintained relatively strong enhancement efficacy. Moderate effects were observed for methional, isoamyl alcohol, and 2,3,5-trimethylpyrazine, while 1-octen-3-ol and 2,3-dimethylpyrazine showed weaker potentiation. Notably, receptor expression studies have demonstrated that methional acted as a positive allosteric modulator (PAM) for human T1R1/T1R3 but functioned as a negative allosteric modulator (NAM) for murine T1R1/T1R3. This species-dependent divergence likely results from methional binding at two distinct sites within the transmembrane domain of T1R1. Amino acid residues at the base of the allosteric pocket facilitate switching between PAM and NAM modes, thereby inducing conformational changes in methional’s binding pose [15]. Additional studies have demonstrated that 3-mercapto-2-methylpentan-1-ol enhanced the rated taste intensity of MSG, whereas it failed to potentiate umami perception under nasal clip conditions. This suggests that the enhancement mechanisms underlying aroma-induced umami potentiation may differ between nasal clip and non-clip experimental paradigms [31]. Further studies have indicated that trans-2-hexenal bonds to the transmembrane domain of human TAS1R2 with multiple critical amino acid residues, being essential for eliciting a sweet taste perception [5]. Similarly, isovaleraldehyde activated the calcium-sensing receptor CaSR in vitro by functioning as a positive allosteric modulator [13].

Regarding the enhancement magnitude, DMS and dimethyl trisulfide were maintained at consistent levels. Increased magnitudes were observed for 1-octen-3-ol, DLE, isoamyl alcohol, and 2,3,5-trimethylpyrazine. In contrast, reduced magnitudes were recorded for methional and 2,3-dimethylpyrazine. Remarkably, sulfur-containing compounds consistently demonstrated a significant enhancement of MSG umami perception—irrespective of nasal clip application.

### 3.2. Homology Modeling of Umami Receptor T1R1/T1R3

**Figure 2:**
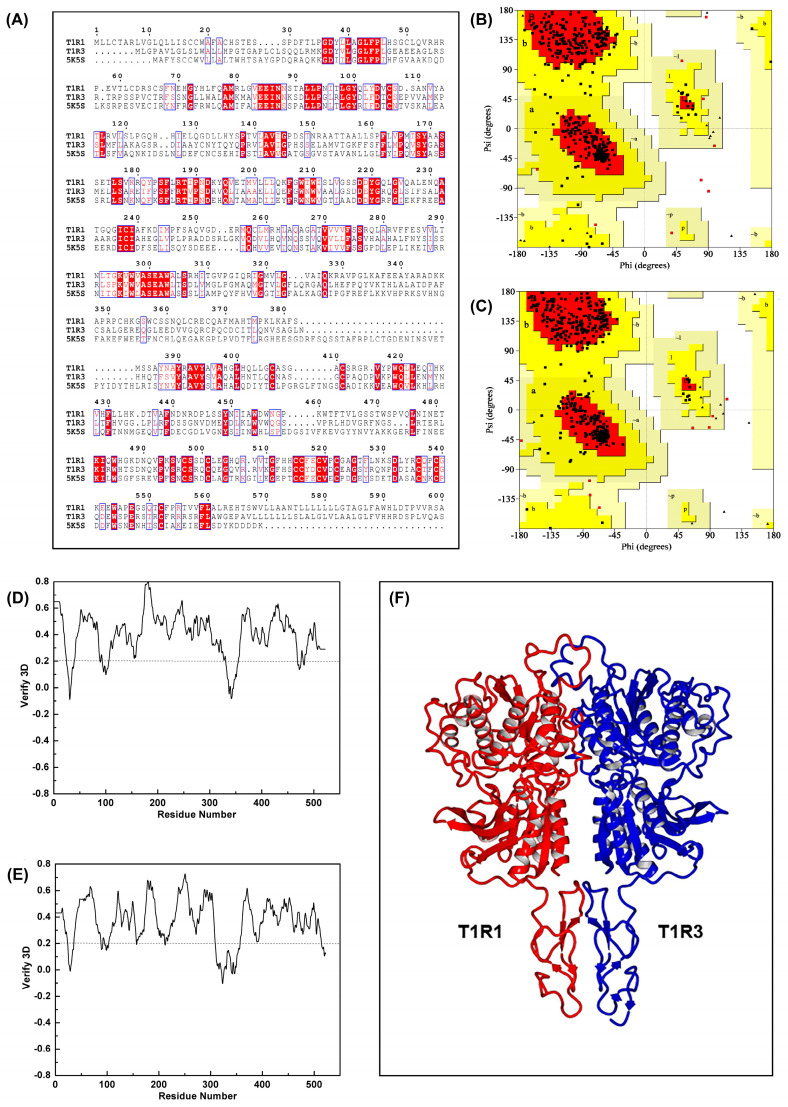
Validation of the T1R1/T1R3 umami receptor homology model. (**A**) Sequence alignment between umami receptor subunits T1R1/T1R3 and template protein 5K5S. (**B**,**C**) Ramachandran plot analysis with the most favored regions (red), additionally allowed regions (yellow), and disallowed regions (unshaded) for the T1R1 and T1R3 subunits, respectively. Conformational regions were denoted as follows: A, core α-helix; a, allowed α-helix; ~a, generous α-helix; B, core β-strand; b, allowed β-strand; ~b, generous β-strand; L, core left-handed α-helix; l, allowed left-handed α-helix; ~l, generous left-handed α-helix; p, allowed ε-region; ~p, generous ε-region. (**D**,**E**) Verify 3D quality scores for the T1R1 and T1R3 subunits, respectively. (**F**) Three-dimensional structure of the T1R1/T1R3 umami receptor.

Five crystal structures were evaluated based on comprehensive metrics including the score, sequence identity, and coverage. Among these, the activated-state crystal structure of the human calcium-sensing receptor extracellular domain (PDB: 5K5S) [32] exhibited the optimal parameters, as shown in Table 2. It is generally accepted that homology modeling can reliably predict protein structures when sequence identity between the target and template exceeds 30% [33]. In this study, an approximately 31% sequence identity was achieved between the 5K5S structure and the umami receptor T1R1/T1R3, surpassing the 30% threshold. The sequence alignment is presented in Figure 2A. Consequently, the 5K5S structure was selected as the template for constructing the T1R1/T1R3 receptor model.

The optimized umami receptor model was evaluated using both the PROCHECK and Verify 3D programs. Ramachandran plot results for the T1R1 and T1R3 subunits are presented in Figure 2B and 2C, respectively. For the T1R1 subunit, 86.20% of the residues occupied the most favored regions, with 0.40% in disallowed regions. The T1R3 subunit showed 89.50% in favored regions and 0.20% in disallowed regions. Consequently, 99.60% of T1R1 residues and 99.80% of T1R3 residues exhibited dihedral angles within acceptable ranges, satisfying the stereochemical energy constraints. The Verify 3D scores for the T1R1 and T1R3 subunits are displayed in Figure 2D,E. Residues scoring ≥0.2 constituted 87.93% and 83.65%, respectively, meeting the compatibility assessment criteria. Integrated analysis of the Ramachandran plots and Verify 3D scores confirmed the structural validity of the protein model. This validated structure served as a reliable template for subsequent investigations. The final T1R1/T1R3 umami receptor model is depicted in Figure 2F.

### 3.3. Modulatory Effects of Aroma Compounds on MSG Binding to the Umami Receptor T1R1/T1R3

#### 3.3.1. Molecular Insights into Umami Enhancement Mechanisms by Aroma Compounds via MD Simulations

**Figure 3:**
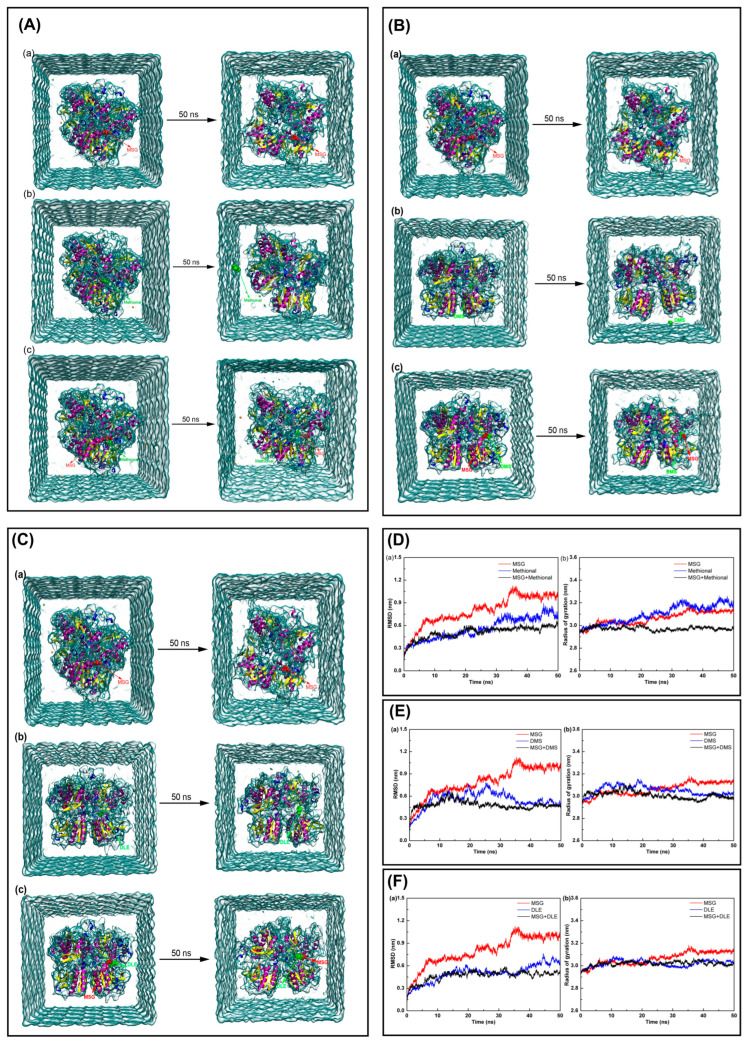
Structural changes and convergence analysis of T1R1/T1R3 receptor systems during molecular dynamics simulation. (**A**–**C**) Structural superimposition before and after 50-ns MD simulation for MSG alone (left), aroma compound alone (middle), and MSG+aroma compound (right) systems with methional (**A**), DMS (**B**), and DLE (**C**). (**D**–**F**) Convergence analysis showing the RMSD values (left) and radius of gyration (right) over simulation time for the methional, DMS, and DLE systems, respectively.

To unfold the molecular mechanism of aroma-induced umami potentiation, DMS, DLE, and methional were chosen for MD simulations based on their optimal umami enhancement in prior sensory tests. Key findings revealed that aroma compounds synergistically stabilized the receptor-ligand binding efficiency. MD simulations (Figure 3A–C) demonstrated the critical phenomenon whereby the structural stability of the MSG–T1R1/T1R3 complex was significantly enhanced upon aroma compound incorporation. In the MSG alone system, stable ligand–receptor binding occurred, while in individual aroma compound systems, methional and DMS progressively dissociated from the binding pocket into the aqueous phase, indicating limited receptor affinity. Strikingly, in ternary complex systems (T1R1/T1R3-MSG-aroma compound), all molecules maintained stable binding within the active site, confirming a synergistic stabilization effect.

Stability analyses (Figure 3D–F) demonstrated superior dynamic stability in ternary complex systems. This was manifested through three vital aspects. Firstly, accelerated convergence was observed. Root mean square deviation (RMSD) analysis revealed that the T1R1/T1R3-MSG-aroma compound systems achieved stabilization within 20–30 ns, markedly faster than the MSG alone system (40 ns). Secondly, reduced structural fluctuations were recorded. The ternary systems exhibited markedly lower RMSD values (0.466–0.558 nm) compared with the MSG alone system (0.992 nm). Thirdly, enhanced structural compactness was achieved. Radius of gyration (Rg) analysis indicated tighter packing in ternary complexes (mean 2.970–2.999 nm) compared with the looser conformation in monoligand systems (3.022–3.178 nm). Thus, aroma compounds function as structural stabilizers.

These discoveries implicate three potential mechanisms by which aroma compounds may enhance umami perception: (a) Allosteric stabilization: aroma compound binding may induce receptor conformational changes, generating an active conformation more favorable for MSG binding; (b) Cooperative binding mode: MSG and aroma compounds likely occupy distinct but interdependent binding sites on the receptor, synergistically enhancing overall binding affinity; (c) Dynamic equilibrium modulation: aroma compounds may reduce the MSG dissociation rates, prolonging receptor activation. This finding, which provides substantial insights, elucidates the molecular basis of aroma-induced umami enhancement. Crucially, aroma compounds not only influence flavor perception via the olfactory pathways, but also directly participate in taste receptor activation, offering a novel molecular explanation for odor–taste interactions. Combined with prior nasal clip sensory data, these results indicate dual enhancement pathways, namely retronasal olfaction and direct receptor modulation.

#### 3.3.2. Aroma Compound-Induced Stabilization of Key Domains in the Umami Receptor T1R1/T1R3

**Figure 4:**
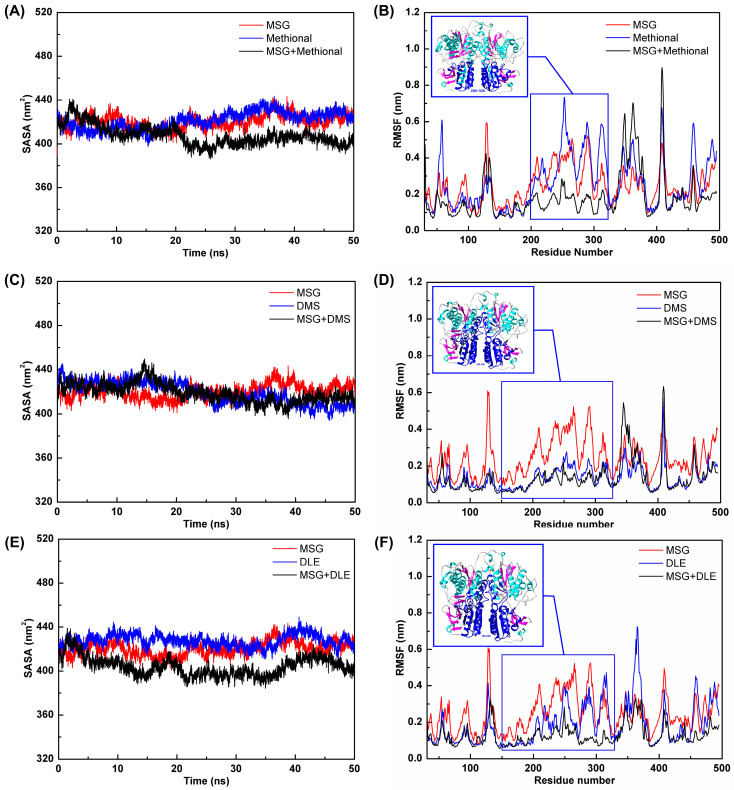
Receptor flexibility analysis during molecular dynamics simulation. (**A**,**C**,**E**) Changes in the solvent accessible surface area (SASA) over simulation time. (**B**,**D**,**F**) Root mean square fluctuation (RMSF) distribution along receptor residues for the methional, DMS, and DLE systems, respectively.

Mechanistic insights into the aroma compound-induced structural stabilization of the T1R1/T1R3 receptor were elucidated through solvent accessible surface area (SASA) and root mean square fluctuation (RMSF) analyses (Figure 4). Results demonstrated that all three aroma compounds promoted receptor stabilization via a shared mechanism: a reduction in solvent accessible surface area and the suppression of flexibility fluctuations within key structural domains.

SASA analysis revealed a critical mechanistic role of aroma compounds that in monoligand systems (T1R1/T1R3-MSG and T1R1/T1R3-aroma compound) elevated the SASA values (410.12–427.26 nm^2^), indicating solvent-exposed receptor conformations. Conversely, ternary complexes exhibited significantly reduced SASA (403.69–412.10 nm^2^), with the most pronounced reduction detected in the T1R1/T1R3-MSG-DLE system (403.69 nm^2^), demonstrating marked synergistic compaction. This SASA decrease reflected weakened receptor–solvent interactions and enhanced structural compaction, which was conducive to maintaining the activated receptor conformation. The observed discrepancy is attributable to the intrinsic physicochemical attributes of DLE and DMS. Due to its bulky, rigid, and strongly hydrophobic architecture, DLE lodges within the hydrophobic core of the receptor in a highly stable manner, elicits domain closure, and sequesters solvent-exposed residues, thereby eliciting a pronounced reduction in SASA. Conversely, DMS, characterized by a compact volume, elevated flexibility, and attenuated hydrophobicity, merely engages transiently with superficial binding loci; its solitary presence fails to establish a persistent occupancy, and the ensuing conformational fluctuations leave the binding-site residues solvent accessible. Specifically, the strong hydrophobic interactions of DLE facilitate its insertion into the hydrophobic cavity, whose dimensions are fully occupied by the macrocyclic framework. This occlusion of residues directly diminishes solvent contact. Moreover, the rigid cyclic scaffold and the presence of double bonds severely restrict conformational freedom, so the entropic penalty upon receptor association is minimized; the resulting robust binding attenuates inter-domain dynamics and consequently decreases the SASA. These mechanistic inferences were also corroborated by MD simulations, which demonstrated that DLE remained stably anchored to the receptor throughout the trajectory.

RMSF analysis further confirmed the stabilization mechanism of aroma compounds. Methional, DMS, and DLE exhibited similar flexibility modulation profiles, with prominent fluctuations localized to residues 150–320. This region, located in the lower lobe of the receptor, likely constitutes a key regulatory domain for ligand-binding stability. Remarkably, ternary systems demonstrated minimal flexibility in this region, which was significantly reduced (*p* < 0.01) compared with the MSG alone systems, achieving optimal stabilization. Aroma compound binding restricted the conformational dynamics of critical domains, attenuating structural fluctuations and prolonging functional MSG engagement. This phenomenon elucidated the molecular basis of taste receptor structural stabilization.

Integrated SASA and RMSF analyses delineated a tripartite structural mechanism for aroma compound-enhanced umami perception: (a) Surface remodeling: aroma compound binding induced receptor surface reorganization, reducing the solvent exposed area and forming a compact protein architecture; (b) Flexibility modulation: flexibility was specifically attenuated in the 150–320 residue region, a putative hinge domain critical for MSG binding stability; (c) Synergistic stabilization: the cooperative action of MSG and aroma compounds optimized receptor stability, preserving the activated conformation while prolonging ligand engagement. These structural transformations provide critical insights that aroma compounds not only bind the receptor, but crucially induce conformational changes that create a structural environment more favorable for MSG binding and activation. This structural cooperativity mechanism probably constitutes a fundamental molecular basis for flavor interactions, offering a theoretical framework for developing novel umami potentiators.

#### 3.3.3. Allosteric Enhancement of MSG–Receptor Interactions by Aroma Compounds

Analysis of the ligand–T1R1/T1R3 interaction patterns revealed the pivotal molecular mechanism underlying the aroma compound-enhanced umami perception, where the presence of aroma compounds significantly strengthened hydrogen bonding interactions between MSG and the receptor, substantially enhancing the binding affinity.

**Figure 5:**
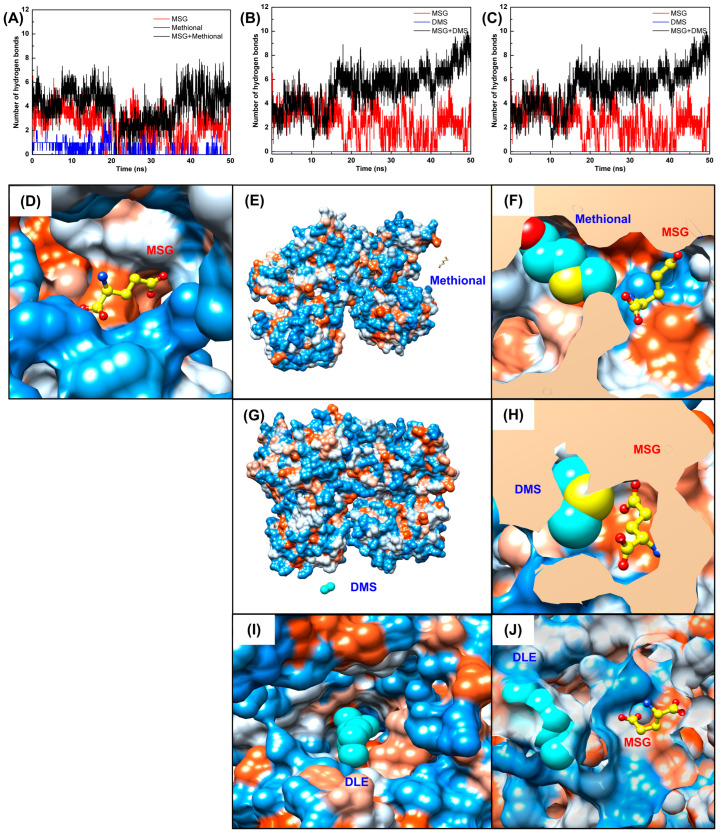
Hydrogen bonding analysis and binding surface visualization. (**A**–**C**) Number of hydrogen bonds between the ligands and receptor over simulation time for the methional, DMS, and DLE systems. (**D**–**J**) Hydrophilic (blue) and hydrophobic (orange) surface binding modes: MSG alone (**D**), individual aroma compounds (**E**,**G**,**I**), and the MSG+aroma compound systems (**F**,**H**,**J**) for methional, DMS, and DLE, respectively.

Hydrogen bonding analysis shown in Figure 5A–C elucidated the core role of aroma compounds, indicating that hydrogen bond network remodeling served as a fundamental synergistic binding mechanism. In monoligand systems, limited hydrogen bonds of MSG were formed, averaging 2.13 bonds, while aroma compounds exhibited minimal hydrogen bonding with methional at 0.09 bonds and DMS/DLE near zero. Ternary complex systems, however, demonstrated significant hydrogen bond augmentation. Specifically, MSG-methional systems established 4.83 bonds (127% increase), MSG-DMS systems formed 7.16 bonds (236% increase), and MSG-DLE systems maintained 5.18 bonds (143% increase). This synergistic potentiation might stem from aroma compound-induced conformational changes that create geometrically favorable configurations for MSG hydrogen bond formation. Zhang et al. discovered via MD simulations that typical umami ligands induced conformational changes in the umami receptor T1R1/T1R3 through intermolecular interactions including hydrogen bonding, van der Waals forces, and electrostatic effects [34]. The umami taste perception of MSG is determined by key molecular properties of its α-carbon including hydrophobicity, size, charge, isoelectric point, chirality, and functional groups on the side chain [35]. Consequently, volatile compounds in the system modulate umami perception by altering molecular interactions between MSG and the taste receptor.

Binding mode analysis in Figure 5D–J revealed that both ligands maintained stable binding within the receptor’s active site in ternary complex systems, where hydrophilic/hydrophobic complementarity governed the binding interface formation. In the MSG alone system, MSG was stably anchored in a hydrophilic pocket (Figure 5D). Upon aroma compound incorporation, colocalized complexes formed with MSG (Figure 5F,H,J), exhibiting spatial proximity between ligands. In this case, direct intermolecular interactions enhanced the MSG-receptor affinity, wherein hydrophobic aroma molecules supplemented the receptor’s hydrophobic surface interactions, while hydrophilic MSG maintained primary pocket binding. This synergy optimized binding domain conformation, establishing the amphiphilic residue network as the structural basis for combinatorial ligand recognition.

**Figure 6:**
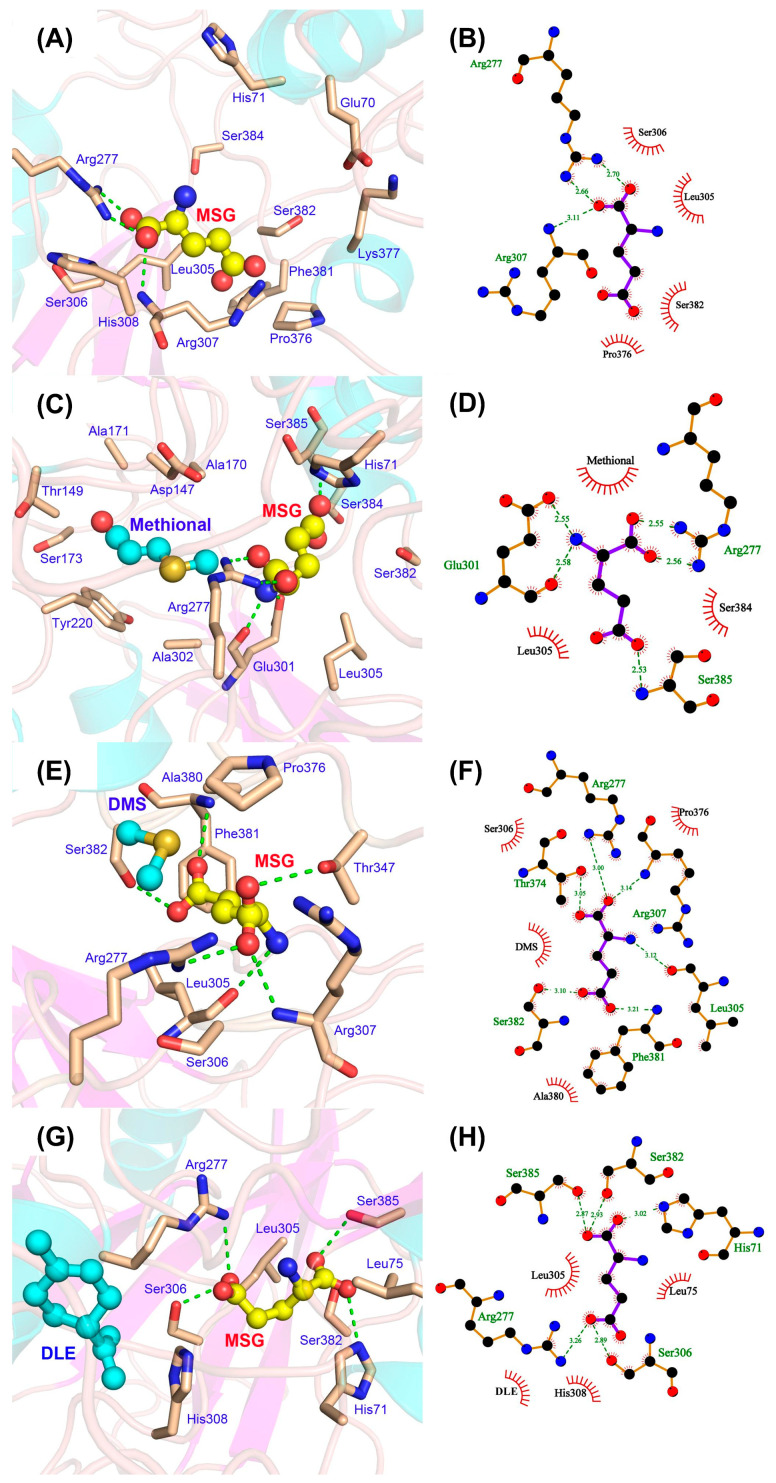
Detailed binding interactions between the ligands and T1R1/T1R3 receptor. Three-dimensional binding conformations (**A**,**C**,**E**,**G**) and two-dimensional interaction diagrams (**B**,**D**,**F**,**H**) for MSG alone and MSG combined with the methional, DMS, and DLE systems, respectively. Green dashed lines represent hydrogen bonds, and red curves represent hydrophobic interactions.

Further binding interaction analyses were conducted to unravel the allosteric regulation mechanism of aroma compounds. Central amino acid residues were identified as cooperatively driving binding site reorganization, thereby enhancing receptor allostery toward specific ligands. When MSG bonded individually to the umami receptor, hydrogen bonds primarily formed with Arg277 and Arg307, resulting in a confined binding pocket. In the presence of aroma compounds, additional residues participated in binding, expanding the interaction interface (Figure 6). Across all systems, Arg277 consistently functioned as the primary hydrogen bond donor, serving as the critical anchor for MSG binding. Aroma compounds engaged with MSG and Arg277 via hydrophobic interactions, facilitating a more stabilized binding microenvironment. Similar results of the importance of Arg277 have likewise been observed in other studies [36,37]. Similar MD studies revealed that limonene addition altered the hydrogen-bonding network in the T1R2/T1R3-sucrose complex, with key residues shifting from Lys65, Glu302, Asp278, and Ser144 to Asp278, Ser144, Asp142, and Asp213. Concurrently, hydrophobic interactions within the system intensified significantly [4].

**Figure 7:**
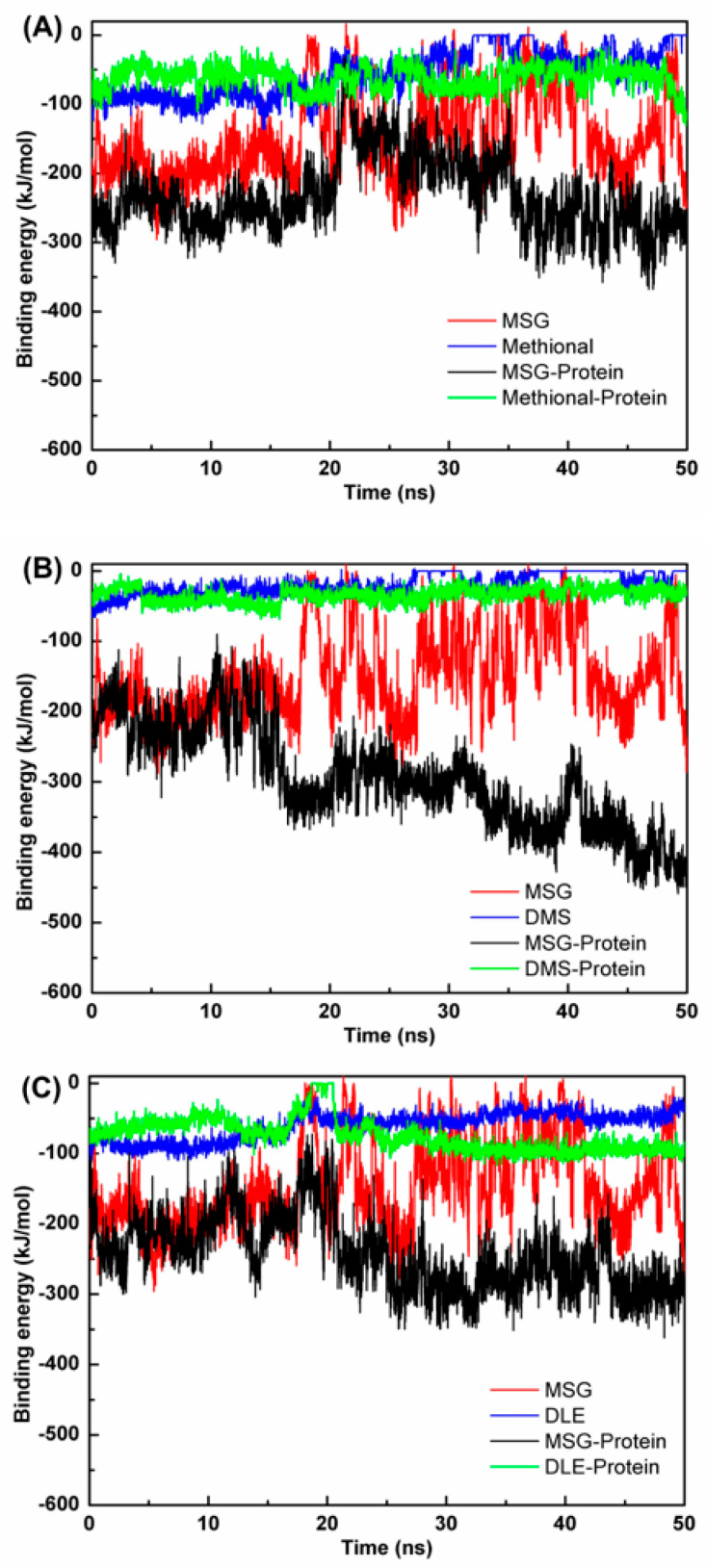
Binding energy profiles during molecular dynamics simulation. Time-dependent binding energies between ligands and T1R1/T1R3 receptor for the (**A**) methional, (**B**) DMS, and (**C**) DLE systems. Lines represent individual compound binding (MSG or aroma compound), MSG-receptor interaction in ternary system (MSG-protein), and aroma compound–receptor interaction in ternary system (aroma-protein).

Binding energy calculations (Figure 7) yielded quantitative thermodynamic evidence. Significantly enhanced binding affinity was observed in ternary complex systems. Specifically, the affinity of MSG for T1R1/T1R3 was −145.42 kJ/mol in the MSG alone system compared with −261.42 kJ/mol (80% increase) in the MSG-methional system, −378.93 kJ/mol (160% increase) in the MSG-DMS system, and −280.99 kJ/mol (93% increase) in the MSG-DLE system. The MSG-DMS system demonstrated the strongest synergistic enhancement effect, which was consistent with its superior performance in sensory evaluation. Conformational rearrangement in the receptor enhanced affinity, thereby amplifying the orthosteric transduction pathway [38]. Complementary research has demonstrated that aroma compounds reduce the binding energy in taste receptors; for instance, limonene decreased the total energy of the T1R2/T1R3-sucrose system from −32.08 kcal/mol to −63.57 kcal/mol [4].

Integrated analyses unveiled the molecular recognition mechanism underlying the aroma compound-mediated allosteric regulation of the umami receptor. This mechanism operates through three distinct pathways. (a) Direct stabilization: aroma compounds bind adjacent to MSG via hydrophobic interactions, forming stabilized ternary complexes. (b) Indirect allostery: aroma compound binding induces receptor conformational changes that optimize the geometry and charge distribution of the MSG binding site. (c) Synergistic network: MSG and aroma compounds cooperatively establish enhanced hydrogen-bonding networks, prolonging receptor activation duration.

In summary, aroma compounds function not only through olfactory channels to modulate flavor perception, but also serve as direct allosteric modulators enhancing taste receptor activation efficiency. Particularly, under nasal clip conditions, this direct receptor-level modulation constitutes the primary mechanism for umami enhancement, providing a theoretical foundation for developing novel umami enhancers targeting allosteric regulation. Furthermore, the molecular dynamics results were strongly corroborated by sensory evaluation. The optimal binding affinity of the DMS system at the molecular level correlated with its superior umami-enhancing efficacy in nasal clip sensory evaluation, validating the computational reliability.

## 4. Conclusions

This study systematically elucidated the mechanism by which aroma compounds enhance MSG umami perception through integrated sensory evaluation and molecular MD simulations. Research findings showed that seafood essence S demonstrated optimal umami enhancement efficacy. Sulfur-containing compounds—specifically methional, DMS, and dimethyl trisulfide—along with DLE and 2,3-dimethylpyrazine were identified as key active constituents. Significantly, under nasal clip conditions eliminating the orthonasal olfactory influence, these compounds persistently enhanced the MSG umami perception, revealing a novel mechanism transcending the traditional cross-modal sensory integration. MD simulations further deciphered the fundamental mechanism. Aroma compounds remodeled the T1R1/T1R3 binding site via allosteric regulation, substantially strengthening the hydrogen bonding interactions between MSG and the receptor (80–160% increase), directly elevating the binding affinity and complex stability. These findings provide the first receptor-level elucidation of aroma compounds directly modulating taste receptor interaction with MSG. This work extends our understanding of cross-modal sensory interactions while establishing a scientific foundation for developing novel umami enhancers based on allosteric principles. Collectively, the study offers a groundbreaking molecular explanation for odor–taste interactions, with significant implications for advancing food flavor modulation technologies.

## Figures and Tables

**Table 1:** Volatile compounds identified in meaty flavorings by GC-MS analysis (μg/g).

No.	CAS	Compound	RI Calc	RI Ref	*m*/*z*	Integral Fragment	ID *	M	B	S
Sulfur-containing compounds										
1	110-66-7	1-Pentanethiol	818	817	104, 42	104	MS, RI	--	2.2639	--
2	513-44-0	1-Propanethiol, 2-methyl-	685	685	41, 90	41	MS, RI	2.0815	--	--
3	24295-03-2	2-Acetylthiazole	1023	1023	43, 99, 127	43	MS, RI	2.7729	--	--
4	NA	2-Furfuryl-2-methyl-3-furyl disulfide	1654	1655	81	81	MS, RI	64.3061	--	--
5	98-02-2	2-Furfurylthiol	913	913	81, 53, 114	81	MS, RI	290.5882	--	--
6	28588-75-2	2-Methyl-3-[(2-methylfuran-3-yl)disulfanyl]furan	1553	1547	113, 226	113	MS, RI	607.1735	--	--
7	28588-74-1	2-Methyl-3-furanthiol	870	870	114	114	MS, RI	99.5886	15.1273	--
8	5271-38-5	2-Methylthioethanol	850	850	61, 92	61	MS, RI	--	--	0.0123
9	1618-26-4	Bis(methylthio)methane	892	892	61, 108	61	MS, RI	--	--	0.0585
10	624-92-0	Dimethyl disulfide	744	744	94, 79	94	MS, RI	0.2441	--	29.6650
11	75-18-3	Dimethyl sulfide	--	521	62, 47	62	MS	--	--	9.9339
12	5756-24-1	Dimethyl tetrasulfide	1215	1215	79, 158	79	MS, RI	--	--	0.0117
13	3658-80-8	Dimethyl trisulfide	975	975	126	126	MS, RI	0.5471	--	1.7146
14	7783-06-4	Hydrogen sulfide	--	--	34	34	MS	0.9203	--	--
15	74-93-1	Methanethiol	--	464	47	47	MS	--	--	0.1530
16	57500-00-2	Methyl furfuryl disulfide	1223	1226	81	81	MS, RI	6.6786	--	--
17	4861-58-9	Thiophene, 2-pentyl-	1168	1170	97, 154	97	MS, RI	434.6640	8.1651	0.1374
18	616-44-4	Thiophene, 3-methyl	772	772	97	97	MS, RI	0.4060	--	--
Pyrazines										
19	14667-55-1	2,3,5-Trimethylpyrazine	1006	1006	122, 42	122	MS, RI	3.7262	--	2.2367
20	15707-24-1	2,3-Diethylpyrazine	1081	1080	121, 136	121	MS, RI	--	--	1.3320
21	5910-89-4	2,3-Dimethylpyrazine	919	919	108, 67	108	MS, RI	--	--	3.3991
22	109-08-0	2-Methylpyrazine	830	830	94, 67	94	MS, RI	--	--	1.1609
Alkenes										
23	18172-67-3	(−)-β-Pinene	976	--	93, 41	93	MS	--	--	0.1352
24	7785-70-8	(+)-α-Pinene	938	937	93	93	MS, RI	--	--	0.0819
25	18368-95-1	1,3,8-p-Menthatriene	1117	1119	119, 134	119	MS, RI	--	--	0.0000
26	586-62-9	1-Methyl-4-(1-methylethylidene) cyclohexene	1093	1094	93, 121, 136	93	MS, RI	--	1.2262	--
27	28634-89-1	2-Thujene	978	978	93	93	MS, RI	--	0.7750	--
28	13466-78-9	3-Carene	1015	1015	93	93	MS, RI	--	5.7671	--
29	104-46-1	Anethole	1294	1289	148	148	MS, RI	--	6550.5112	--
30	79-92-5	Camphene	954	954	93, 121	93	MS, RI	--	0.3364	--
31	87-44-5	Caryophyllene	1441	1441	93, 133	93	MS, RI	0.3445	128.6968	--
32	1139-30-6	Caryophyllene oxide	1614	1613	93	93	MS, RI	--	0.5720	--
33	18252-46-5	Cis-α-bergamotene	1428	1428	119, 93	119	MS, RI	--	103.4251	--
34	3856-25-5	Copaene	1391	1391	119, 105, 161	119	MS, RI	--	63.5100	--
35	5989-27-5	D-Limonene	1034	--	68, 93	68	MS	0.8658	27.2089	5.6548
36	6753-98-6	Humulene	1476	1477	93	93	MS, RI	--	13.0814	--
37	78-70-6	Linalool	1102	1102	71, 93	71	MS, RI	--	4.2015	--
38	58319-06-5	Sesquithujene	1414	1417	119, 93	119	MS, RI	--	3.2553	--
39	562-74-3	Terpinen-4-ol	1186	1186	71, 111	71	MS, RI	--	0.8337	--
40	546-80-5	Thujone	1112	1112	81, 110	81	MS, RI	--	0.7441	--
41	13474-59-4	Trans-α-bergamotene	1449	1450	119, 93	119	MS, RI	--	103.8404	--
42	495-60-3	Zingiberene	1506	1506	119, 93	119	MS, RI	--	53.5610	--
43	469-61-4	α-Cedrene	1436	1436	119	119	MS, RI	--	4.6260	--
44	502-61-4	α-Farnesene	1513	1513	93	93	MS, RI	--	3.2607	--
45	495-61-4	β-Bisabolene	1520	1520	69, 93	69	MS, RI	--	20.1507	--
46	515-13-9	β-Elemene	1405	1405	93, 81, 107	93	MS, RI	--	13.1540	--
47	127-91-3	β-Pinene	982	982	93	93	MS, RI	--	2.3582	--
48	17066-67-0	β-Selinene	1510	1509	105	105	MS, RI	--	1.1195	--
49	20307-83-9	β-Sesquiphellandrene	1538	1536	69, 93	69	MS, RI	--	14.9319	--
50	99-85-4	γ-Terpinene	1064	1064	93, 136	93	MS, RI	--	3.0459	0.0401
51	20307-84-0	δ-Elemene	1350	1351	121, 93, 136, 161	121	MS, RI	--	23.6534	--
52	106-99-0	1,3-Butadiene	--	400	54, 39	54	MS	--	--	0.0988
53	872-05-9	1-Decene	992	992	43, 56, 70	43	MS, RI	--	--	0.0933
54	112-41-4	1-Dodecene	1196	1195	69, 43	69	MS, RI	--	--	0.0746
55	124-11-8	1-Nonene	892	892	43, 56	43	MS, RI	0.8351	1.7049	--
56	627-20-3	2-Pentene, (Z)-	--	510	55, 70	55	MS	--	--	0.9789
57	691-37-2	4-Methyl-1-Pentene	--	553	43, 56	43	MS	--	--	0.2348
58	100-42-5	Styrene	894	894	104, 78	104	MS, RI	--	0.7356	--
59	17699-05-7	α-Bergamotene	1428	1428	93, 119	93	MS, RI	0.0505	--	--
Alcohols										
60	928-96-1	(Z)-hex-3-en-1-ol	855	855	67, 82, 41, 55	67	MS, RI	--	--	2.2041
61	1565-80-6	1-Butanol, 2-methyl-, (S)-	--	--	56, 41	56	MS	--	--	0.3792
62	15250-22-3	1-Octanol, 2,7-dimethyl-	--	144	43, 58	43	MS	--	--	0.1527
63	3391-86-4	1-Octen-3-ol	982	982	57	57	MS, RI	5.0144	--	10.8963
64	928-97-2	3-Hexen-1-ol, (E)-	855	855	67, 41	67	MS, RI	--	--	0.0231
65	123-51-3	Isoamyl alcohol	--	730	55, 70	55	MS	--	--	2.9690
Ketones										
66	600-14-6	2,3-Pentanedione	--	698	43, 57, 100	43	MS	0.6208	--	--
67	78-93-3	2-Butantone	--	609	43, 72	43	MS	1.4412	5.7114	--
68	693-54-9	2-Decanone	1195	1195	58, 43, 71	58	MS, RI	0.4498	--	--
69	110-43-0	2-Heptanon	889	889	43, 58	43	MS, RI	--	--	0.1451
70	821-55-6	2-Nonanone	1093	1094	58	58	MS, RI	--	0.4057	0.0206
71	111-13-7	2-Octanone	992	992	43, 58	43	MS, RI	1.0648	--	--
72	107-87-9	2-Pentanone	--	697	43, 86	43	MS	1.1319	4.2671	--
73	563-80-4	3-Methyl-2-butanone	--	653	43, 86	43	MS	--	--	0.2921
74	96-22-0	3-Pentanone	700	700	57, 86	57	MS, RI	0.8016	3.3342	--
75	2216-87-7	3-Undecanone	1290	1283	141, 57, 72	141	MS, RI	--	2.1575	--
76	30086-02-3	Trans,trans-3,5-octadien-2-one	1097	1098	95, 43	95	MS, RI	--	--	0.0048
Furan(one)s										
77	104-61-0	2(3H)-Furanone, dihydro-5-pentyl-	1363	1363	85, 44	85	MS, RI	--	--	0.0115
78	3188-00-9	Dihydro-2-methyl-3-furanone	808	808	43, 72, 100	43	MS, RI	12.5517	--	0.1365
79	1197-40-6	Furan, 2,2′-methylenebis-	1088	1089	91, 148	91	MS, RI	0.7623	--	--
80	930-27-8	Furan, 3-methyl-	--	614	82, 53	82	MS	2.6717	--	--
81	3777-69-3	Furan, 2-pentyl	994	994	81	81	MS, RI	0.6586	--	--
Aldehydes										
82	25152-84-5	(E,E)-2,4-Decadienal	1324	1324	81	81	MS, RI	--	--	0.0143
83	557-48-2	2,6-Nonadienal, (E,Z)	1156	1156	41, 70	41	MS, RI	--	--	0.3315
84	3913-81-3	2-Decenal, (E)-	1268	1268	70, 55	70	MS, RI	0.0544	1.1817	--
85	13019-16-4	2-Octenal, 2-butyl-	1379	1378	55	55	MS, RI	--	0.6627	--
86	590-86-3	3-Methylbutanal	--	655	44, 58, 71	44	MS	--	--	0.0901
87	6728-31-0	4-Heptenal, (Z)-	902	902	41, 55, 68, 84	41	MS, RI	--	--	0.2251
88	122-03-2	4-Isopropylbenzaldehyde	1252	1252	133, 105	133	MS, RI	--	3.8862	--
89	100-52-7	Benzaldehyde	966	965	105, 77, 51	105	MS, RI	--	6.8549	0.3813
90	112-31-2	Decanal	1208	1208	57	57	MS, RI	0.1426	3.0725	--
91	56-82-6	DL-Glyceraldehyde	--	--	91, 61	91	MS	--	--	0.0174
92	98-01-1	Furfural	835	835	95, 39	95	MS, RI	--	--	0.2524
93	111-71-7	Heptanal	903	903	57	57	MS, RI	0.1651	1.6242	--
94	66-25-1	Hexanal	801	801	44, 56	44	MS, RI	0.2643	1.2348	0.0580
95	3268-49-3	Methional	909	909	48, 104	48	MS, RI	--	--	8.5728
96	124-19-6	Nonanal	1105	1105	57	57	MS, RI	1.5959	9.2310	0.0256
97	124-13-0	Octanal	1004	1004	43, 57, 84	43	MS, RI	0.1715	0.8794	--
98	124-25-4	Tetradecanal	1579	1579	82	82	MS, RI	--	39.9319	--
Esters										
99	123-92-2	1-Butanol, 3-methyl-, acetate	878	878	43, 55, 70	43	MS, RI	--	--	0.0938
100	110-45-2	1-Butanol, 3-methyl-, formate	792	792	55, 70	55	MS, RI	--	--	0.0827
101	868-57-5	2-Methylbutanoic acid methyl ester	771	771	88, 57	88	MS, RI	--	--	0.0693
102	3681-82-1	3-Hexen-1-ol,1-acetate, (3E)-	1008	1008	67, 82	67	MS, RI	--	6.7108	--
103	2035-99-6	3-Methylbutyl octanoate	1453	1452	70	70	MS, RI	--	--	0.0077
104	140-11-4	Acetic acid benzyl ester	1170	1170	108, 91, 150	108	MS, RI	--	1.4869	--
105	112-14-1	Acetic acid octyl ester	1212	1213	43, 56, 70	43	MS, RI	--	46.0491	--
106	123-86-4	Acetic acid, butyl ester	816	816	43, 56	43	MS, RI	0.8136	--	0.0524
107	105-54-4	Butanoic acid, ethyl ester	803	803	71, 43, 88	71	MS, RI	0.3658	0.9472	0.5675
108	623-42-7	Butanoic acid, methyl ester	723	723	74, 43	74	MS, RI	1.1396	3.4593	0.2654
109	589-75-3	Butyl caprylate	1388	1388	56, 145	56	MS, RI	0.4217	--	--
110	110-38-3	Decanoic acid, ethyl ester	1395	1395	88, 101	88	MS, RI	6.4838	5.0649	--
111	110-42-9	Decanoic acid, methyl ester	1327	1327	74, 87, 143	74	MS, RI	--	--	0.0141
112	616-38-6	Dimethyl carbonate	--	620	45, 59	45	MS	--	--	--
113	106-33-2	Dodecanoic acid, ethyl ester	1594	1594	88, 101	88	MS, RI	--	1.4096	--
114	141-78-6	Ethyl acetate	--	615	43	43	MS	4.3639	--	0.2620
115	93-89-0	Ethyl benzoate	1178	1177	105, 77, 122	105	MS, RI	--	1.3972	--
116	105-37-3	Ethyl propionate	726	726	57, 77, 43	57	MS, RI	--	--	0.0315
117	142-92-7	Hexyl acetate	1014	1014	43, 56	43	MS, RI	--	11.8858	--
118	554-12-1	Methyl propionate	--	622	57, 88	57	MS	--	2.3802	0.1936
119	106-32-1	Octanoic acid, ethyl ester	1197	1197	88, 101	88	MS, RI	43.0167	2.2618	0.0115
120	111-11-5	Octanoic acid, methyl ester	1125	1125	74, 87	74	MS, RI	3.0726	0.3832	0.2696
121	80-26-2	Terpinyl acetate	1359	1359	121, 93, 136	121	MS, RI	--	10.8402	--
122	124-06-1	Tetradecanoic acid, ethyl ester	1793	1793	88, 101	88	MS, RI	--	5.6830	--
123	102-76-1	Triacetin	1349	1350	43, 103, 145	43	MS, RI	--	--	0.0132
ethers										
124	140-67-0	Estragole	1205	1204	148	148	MS, RI	--	109.1040	--
Acids										
125	334-48-5	Decanoic acid	1367	1367	60, 73, 129	60	MS, RI	0.7768	--	--
126	143-07-7	Dodecanoic acid	1563	1562	73	73	MS, RI	--	1.8547	--
127	124-07-2	Octanoic acid	1175	1175	60, 73	60	MS, RI	2.3087	1.4629	--
Others										
128	95-47-6	1,2-Xylene	896	896	91, 106	91	MS, RI	0.7234	--	0.0595
129	91-57-6	2-Methylnaphthalene	1308	1308	142	142	MS, RI	--	2.4538	--
130	100-41-4	Ethylbenzene	864	864	91, 106	91	MS, RI	0.8535	0.9918	--
131	527-84-4	o-Cymene	1029	1029	119	119	MS, RI	--	8.9806	0.1382
132	108-88-3	Toluene	767	767	91	91	MS, RI	1.3531	2.4152	--

* Identification method: MS, identified by mass spectral matching with the NIST14 library; RI, identified by a retention index comparison with the literature values.

**Table 2:** Homology modeling template selection results for the T1R1 and T1R3 subunits.

Crystal Structure	T1R1				T1R3			
	Score	E Value	Identity	Cover	Score	E Value	Identity	Cover
5K5S	299	2 × 10^−90^	31.59%	64%	283	2 × 10^−84^	31.05%	64%
5X2M	267	4 × 10^−80^	34.75%	55%	204	5 × 10^−57^	30.17%	54%
6N52	266	4 × 10^−76^	26.85%	95%	124	1 × 10^−28^	25.71%	75%
5FBH	239	8 × 10^−69^	31.02%	54%	228	1 × 10^−64^	30.11%	55%
2E4U	164	2 × 10^−42^	26.22%	62%	166	6 × 10^−43^	26.00%	63%

## Data Availability

The original contributions presented in the study are included in the article, further inquiries can be directed to the corresponding author.
